# The Role of the RGD Motif of the IdeC Protein in *Streptococcus canis* in Adhesion and Invasion

**DOI:** 10.3390/microorganisms14040919

**Published:** 2026-04-18

**Authors:** Saoirse Walsh, Alba Garay-Álvarez, Manfred Rohde, Markus Keller, Juan Hermoso, Simone Bergmann, Marcus Fulde

**Affiliations:** 1 Institute of Microbiology and Epizootics, School of Veterinary Medicine, Freie Universität Berlin, 141630 Berlin, Germany; s.walsh@fu-berlin.de; 2 Department of Crystallography and Structural Biology, Institute of Physical-Chemistry “Blas Cabrera”, CSIC, 28006 Madrid, Spain; agaray@iqf.csic.es (A.G.-Á.);; 3 Helmholtz Centre for Infection Research, 38124 Braunschweig, Germany; 4 Institute of Novel and Emerging Infectious Diseases, Friedrich-Loeffler-Institut, Federal Research Institute for Animal Health, Insel Riems, 17493 Greifswald, Germany; markus.keller@fli.de; 5 Institut für Mikrobiologie, Technische Universität Braunschweig, 38106 Braunschweig, Germany; 6 Institute of Microbiology, University of Veterinary Medicine Hannover, Foundation, Bemeroder Straße 31, 30559 Hannover, Germany

**Keywords:** adhesion, invasion, RGD motif, virulence factor, cysteine protease

## Abstract

*Streptococcus canis* is an opportunistic pathogen that colonises the mucosal surfaces and skin of its host. Though predominantly a veterinary pathogen affecting cats and dogs, *S. canis* has also been identified as the causative agent in severe human disease. IdeC is a secreted cysteine protease of *S. canis* that has a high specificity for IgG, cleaving at the hinge region. We show here that the protein binds back to the surface of the bacteria. Additionally, the protein contains a conserved Arg-Gly-Asp (RGD) motif, the minimal peptide sequence required for integrin binding. Several bacterial proteins containing RGD motifs have been implicated in adhesion and invasion of host cells. This RGD motif along with the ability of IdeC to bind back to the bacterial surface after secretion is the basis for this study into a potential secondary function of IdeC in adhesion and/or invasion. We used protein-coated latex beads to investigate the interaction of IdeC with epithelial and endothelial cells and, further, the extent to which the RGD motif is involved in this interaction by utilising an RGD->RGE recombinant protein. We also report here that the deletion of IdeC in *S. canis* results in a significant reduction in invasion into epithelial cells.

## 1. Introduction

*Streptococcus canis* (*S. canis*) is a Gram-positive, ß-haemolytic, opportunistic pathogen [1] belonging to Lancefield group G or C [2]. Though originally thought to affect only dogs and cows, *S. canis* is a multi-host pathogen, having been isolated from a broad range of animals, including foxes, mink and rabbits. Nevertheless, cats and dogs are the predominant hosts [1,3,4]. *S. canis* colonises the mucosal surfaces and skin of the host, but is also an opportunistic pathogen. Clinical presentations include infections of the mucosa and skin, septicaemia, endocarditis, and necrotising fasciitis [1].

Though *S. canis* has been isolated from a wide range of hosts, it is not as well studied as other streptococcal species. However, based on the zoonotic potential of this pathogen, along with the acquisition of antimicrobial resistance (AMR) that has now been shown, it is increasingly recognised as a potential threat [5,6,7]. The virulence factors of *S. canis* are not well characterised, although some have been described, including the *S. canis* M Protein, SCM [8,9,10], and an arginine deiminase system [11].

Clinical manifestation suggests that similar virulence factors may exist in *S. canis* as in *Streptococcus pyogenes* (*S. pyogenes*). Previous studies describe genome comparisons as a starting point for investigating possible virulence factors of *S. canis* [6,12]. Cysteine proteases have been identified as potent virulence factors in many bacteria, including various streptococcal species. IdeS (also called Mac-1) is a cysteine protease of *S. pyogenes* that has been demonstrated to cleave human immunoglobulin G (IgG), thereby inhibiting phagocytosis [13,14]. In our previous work we described the cysteine protease IdeC (Immunoglobulin G-degrading enzyme of *S.* *c**anis*), identified based on a sequence similarity to IdeS [15]. IdeC is a highly specific endopeptidase that cleaves feline, canine and human IgG in the hinge region. IdeC also contains an Arg-Gly-Asp (RGD) motif [15].

The RGD motif is an amino acid motif involved in integrin binding [16]. This motif is found in many proteins involved in mediating cell-to-cell interaction [17]. The engagement of extracellular matrix (ECM) proteins such as fibronectin, vitronectin and fibrinogen with integrins is primarily mediated by RGD motifs present within the ECM proteins [18]. Integrin interaction with ECM proteins results in the activation of intracellular signalling pathways via recruitment of the signalling molecules to the integrin engagement/clustering site [19]. This binding and subsequent clustering of integrins has been demonstrated to be necessary for the activation of integrin signalling as well as invasion into cells by bacteria [20]. In proteins such as human fibronectin, the RGD motif has been revealed to be located on an outward-facing loop, making it easily accessible to a single integrin molecule [21]. The position of the RGD motif, its 3D structure, flanking residues, etc., are factors in the interaction that may occur between the RGD motif and integrin receptors. Several bacterial proteins have been shown to have a role in human infection mediated by an RGD motif, for example, CagL of *Helicobacter pylori* [22], Pertactin of *Bordetella pertussis* [23], and SfbI/F1 of *S. pyogenes* [24,25]. Indeed, the IdeS protein that shares a high sequence identity with IdeC also contains the RGD motif, which has been demonstrated to mediate integrin interaction [26].

This study aims to determine whether the previously described cysteine protease IdeC is a ‘moonlighting’ protein with a second role, in adhesion or invasion. The IgG-cleaving properties of IdeC have been described, but a secondary function will be investigated here based on the presence of an integrin-binding RGD motif and the ability of the protein to bind to the bacterial surface after secretion.

## 2. Materials and Methods

### 2.1. Bacterial Strains and Growth Conditions

The *Streptococcus canis* strains G361 [27] and Sc957 (isolated from skin infection in a canine, during routine diagnostics at Freie Universität Berlin, Germany) were routinely grown in Todd–Hewitt broth (THB) at 37 °C. Strains carrying the pGhost9 plasmid or its derivatives were grown in the same medium supplemented with 200 µg/mL erythromycin at 28 °C. The *Escherichia coli* strain M15 pREP was grown at 37 °C in lysogeny broth (LB) supplemented with 25 µg/mL kanamycin. After transformation with the pQE30 plasmid, M15 pREP pQE30 was grown in LB supplemented with 100 µg/mL ampicillin and 25 µg/mL kanamycin.

### 2.2. Analysis of IdeC Amino Acid Sequence

The sequence for IdeC was taken from the NCBI entry, ‘Immunoglobulin G-endopeptidase (IdeS)/Mac [Streptococcus canis FSL Z3-227]’, accession: EIQ82374. The program SignalP 6.0 (https://services.healthtech.dtu.dk/services/SignalP-6.0/ (accessed on 11 June 2024)) was used to analyse the protein for the presence of a signal sequence and locate it.

### 2.3. Recombinant Protein Expression and Purification

Genomic DNA was extracted from an overnight bacterial culture using the QIAGEN QIAamp DNA Mini Kit. The IdeC protein was amplified from gDNA using the IdeC-sigseq BamH1 (5′-GCTGGATCCGACAACATCGA-3′) and IdeC HindIII (5′-GGGAAGCTTTTAGTTTGATGCC-3′) PCR primers. The PCR product was analysed by agarose gel electrophoresis, on a 1.5% gel. The PCR product was then purified using the QIAGEN QIAquick PCR Purification Kit. The purified PCR product and the pQE30 plasmid were then digested using the BamHI and HindIII restriction enzymes; digestion was carried out at 37 °C for 1 h. The digested PCR product was ligated into the digested plasmid using T4 ligase at 4 °C overnight. The pQE30_IdeC plasmid was then transformed into the *E. coli* strain M15 pREP. The M15 pREP pQE30_IdeC was grown to OD_600_ = 0.5 at 29 °C, IPTG was added to a final concentration of 1 M, and the culture was grown for a further 4 h at 29 °C. Cells were harvested by centrifugation and lysed using a 1 mg/mL lysozyme solution. The protein was then purified by nickel chromatography on Protino Ni-TED 1000 columns. Proteins were run on 15% SDS gel to confirm that purification was successful. Thermo Scientific Slide-A-Lyzer Dialysis Cassettes with a 10,000 molecular-weight cutoff were used to exchange the elution buffer, so proteins could be stored in 1 × PBS.

### 2.4. Preparation of Recombinant Proteins

To prepare a recombinant IdeC protein (rIdeC) with an amino acid replacement of the RGD to RGE, inverse PCR was performed on the pQE30_IdeC construct using the PCR primers RGEfor (5′-CAGGTATTTACAAGAGGAGAACAAAGTAAACTATTA-3′) and RGErev (5′-TTTACTTTGTTCTCCTCTTGTAAATACCTGATCAAA-3′). The PCR product was purified and digested with the Dpn1 restriction enzyme for 1 h at 37 °C, before ligation with T4 ligase. pQE30IdeCRGE was transformed into M15 pREP, and the protein was purified as described above.

### 2.5. IgG Cleavage Assay

To demonstrate the IgG cleavage abilities of IdeC_RGE_, 3 µg of purified rIdeC_RGE_ was incubated with 3 µg purified feline IgG (Jackson Immuno Research, Westgrove, PA, USA) for 4 h at 37 °C. 4× Laemmli sample buffer with dithiothreitol (DTT) was added to the reaction mix and it was denatured at 95 °C for 10 min. The reaction mix was run on 15% SDS gel to visualise results. Precision Plus Protein™ All Blue Pre-stained Protein Standard was used to determine the molecular weight of bands (BioRad, Hercules, CA, USA, catalogue number 1610373).

### 2.6. Preparation of the IdeC-Coated Latex Beads

For experiments involving protein-coated beads, SIGMA (Sigma-Aldrich, Saintlouis, MO, USA) fluorescent carboxyl-modified latex beads or SIGMA latex beads were used. In total, 108 beads were washed 3 times in 1 × PBS, and then coated with 1000 µg protein overnight at 4 °C, with rotation. In the case of carboxyl-modified beads, EDAC was used as a chemical linker at a final concentration of 0.15–2.6 mM. Finally, beads were washed three times in 1 × PBS. Successful coating of the beads was confirmed by SDS-Page.

### 2.7. Cell Culture and Bead Internalisation Assay

All cell lines used in this study (Table 1) were incubated at 37 °C and 5% CO_2_. The MEF cell lines were kindly gifted by Dr. Markus Keller of the Friedrich-Loeffler Institut. The A549 cells were provided by Dr. Thomas Meyer of the Max Planck Institute for Medical Research.

**Table 1:** Sources of cell types used in this study.

Cell Line	Cell Type	Source	Medium
A549	Hypotriploid alveolar basal epithelial cells	Max Planck Institute, Meyer Lab	DMEM 10% FCS
HUVEC	Primary human endothelial cells from the vein of the umbilical cord	PromoCell	Endothelial cell culture medium
MEF	Fibroblasts prepared from mouse embryo	Friedrich-Loeffler Institut, Keller Lab [28]	F12/DMEM 10% FCS
MEF β_3_ −/−	Fibroblasts prepared from mouse embryo with β_3_ subunit deletion	Friedrich-Loeffler Institut, Keller Lab [28]	F12/DMEM 10% FCS
MEF α_V_ −/− β_3_ +/−	Fibroblasts prepared from mouse embryo with α_V_ subunit deletion and a heterologous β3 subunit deletion	Friedrich-Loeffler Institut, Keller Lab [28]	F12/DMEM 10% FCS
MEF α_V_β_3_ −/−	Fibroblasts prepared from mouse embryo with both α_V_β_3_ integrin subunits deleted	Friedrich-Loeffler Institut, Keller Lab [28]	F12/DMEM 10% FCS

Bead assays were carried out as previously described by Kaur et al. [29]; cells were seeded onto coverslips in 24-well plates and grown to ~80% confluency. Latex beads (Sigma-Aldrich, Saintlouis, MO, USA) were coated in protein by incubation with purified recombinant protein overnight, rotating at 4 °C. Beads were then washed three times with 1 × PBS. Protein-coated beads were suspended in endothelial cell culture medium and added to cell monolayers at an excess of 100 beads per cell. Cells were incubated with beads at 37 °C, 5% CO_2_, for various time periods (30 min, 1 h, 2 h). After incubation, monolayers were washed 6 times with 1 × PBS before fixing with 4% paraformaldehyde. To ensure non-specific binding of beads did not occur, bovine serum albumin (BSA)-coated latex beads were used in the same assay as a control.

### 2.8. Fluorescence Staining and Confocal Microscopy

Coverslips with fixed cells were first blocked for 30 min with PBS containing 5% foetal calf serum (FCS). For visualisation of cell nuclei and actin cytoskeletons, DAPI and Phalloidin were used, respectively. In monolayers treated with fluorescent beads, no further staining was performed. For monolayers treated with non-fluorescent beads, extracellular beads were stained using rabbit polyclonal anti-IdeC IgG followed by anti-rabbit Alexa Fluor 488-conjugated IgG. Mounted samples were viewed and analysed using the Leica DMI6000 B microscope with a 63× objective lens, and adherent/associated beads were counted. Bead counts were performed as follows: 6 fields of view (FOVs) per slide were examined. FOVs were selected based on a similar number/distribution of cells, and then the number of beads in clusters, the number of single beads and the number of clusters were recorded per FOV.

All images were deconvolved using Leica Application Suite 4,0. Images were brightened identically using IrfanView 64-bit software.

### 2.9. Preparation of 957ΔideC Strain

To prepare an IdeC deleted strain, the *ideC* gene of the *S. canis* strain 957, along with the upstream and downstream regions, was amplified using the PCR primers IdeCup (5′-CCCCCATGGCACCTGATAATATCGCCTC-3′) and IdeCdw (5′-GGGCCATGGACGAGCATCATTACTGGG-3′). The plasmid pGhost9::ISS1 was amplified using the PCR primers pGhost9-dISS1 F (5′-GGGCCATGGGCTCCTTGGAAGCTGTCAG-3′) and pGhost9-dISS1 R (5′-CCCCCATGGGGTACCCAATTCGCCCTATA-3′). Both PCR products were purified and digested with Nco1 restriction enzyme, before being ligated using T4 ligase at 4 °C overnight. pGhost9_*ideC*up+dw was transformed into *E. coli* EC101 using the BIO-RAD MicroPulser (1.8 kV). The pGhost9_ideCup+dw construct was purified from *E. coli* EC101 using QIAGEN QIAprep Spin Miniprep Kit and the *ideC* gene was removed by inverse PCR using the primers IdeCdel-fw (5′-GGGGATATCAGAGCCCAAGACATGCT-3′) and IdeCdel-rv (5′-CCCCTATAGATGCTGGACTTAGGCAT-3‘). The PCR product was purified and digested with EcoRV before ligation with T4 ligase. The pGhost9Δ*ideC* construct was transformed into *E. coli* EC101. The pGhost9Δ*ideC* construct was purified from EC101 and transformed via electroporation into 957 using the BIO-RAD MicroPulser (1.8 kV).

To induce allelic replacement, a single colony of 957 pGhost9Δ*ideC* was inoculated into THB 200 µg/mL erythromycin and grown overnight at 28 °C. This culture was sub-cultivated and grown to OD600 0.2 at 28 °C; this culture was left at 37 °C overnight. A serial dilution was performed on the culture; it was plated on Todd–Hewitt agar (THA) containing 200 µg/mL erythromycin and incubated overnight at 28 °C. A single colony was inoculated into THB without erythromycin and grown overnight at 28 °C. The culture was serially diluted and plated on THA without erythromycin and grown overnight at 37 °C. Single colonies were picked and tested on THA with and without erythromycin at 37 °C. Colonies that were erythromycin-sensitive were checked for the presence of the *ideC* gene using the IdeCup and IdeCdw PCR primers.

### 2.10. Bacterial Infection Assay

Bacterial infections were performed in the A549 cell line. Cells were seeded in 24-well plates and grown to 90% confluence. *S. canis* strains were grown to mid-log phase in THB (OD_600_ ~ 0.3). Monolayers were washed 3 times with PBS before cell culture media was replaced with media containing *S. canis* at a multiplicity of infection (MOI) of 1:10 (10 bacteria for each cell). Bacterial inoculates were standardised based on absorbance at OD_600_, CFU counts were used to confirm bacterial counts, and cultures were vortexed prior to application to monolayers to disrupt any bacterial aggregates. The plate was incubated at 37 °C and 5% CO_2_ for 2 h to allow for bacterial adhesion. Monolayers were washed six times to remove non-adherent bacteria. After washing, 0.01% Triton X-100 was added to lyse cells. Cell lysates were serially diluted in PBS, plated on THA and incubated overnight to count the colony-forming units (CFU).

To determine levels of internalised bacteria, steps were performed as above, but extracellular bacteria were inactivated after removal of non-adherent bacteria by further incubation of 40 min at 37 °C, 5% CO_2_, in DMEM containing 150 µg/mL erythromycin. Afterwards, wells were washed 6 times with PBS and then lysed with 0.01% Triton X-100. As above, cell lysates were serially diluted, plated on THA and grown overnight to allow CFU determination.

### 2.11. Electron Microscopy

For the labelling of rebound IdeC on the *S. canis* bacterial surface, the protocol by Bergmann et al. 2017 was applied [30]. In short, S. canis cells were washed with 1 × PBS, incubated with a 1:50 dilution in PBS of protein A-purified rabbit IgG protein against IdeC (~75 µg IgG protein) for 1 h at 37 °C. After several washing steps with PBS, bound IgG was visualised by incubation with protein A-gold/protein G-gold nanoparticles (ratio 1:1, 15 nm in size) for 30 min at 37 °C. As a control, specific IdeC, IgG antibodies were incubated with IdeC protein before incubation with S. canis. After washing with PBS and TE buffer (10 mM TRIS, 2 mM EDTA, pH 6.9), samples were placed onto butvar-coated copper grids and air-dried. Grids were attached to adhesive carbon tape on aluminium stubs and samples were examined in a Zeiss Merlin (Oberkochen, Germany), applying the Everhart-Thornley HESE2 secondary electron detector at an acceleration voltage of 10 kV. Images were taken with the Zeiss SmartSEM software version 5.05.

### 2.12. Bright-Field Microscopy

Liquid cultures of Sc957 and Sc957∆ideC were viewed and analysed using the Leica DMI6000 B microscope with a 63× objective lens.

### 2.13. Statistical Analysis

All statistical analyses were performed using GraphPad Prism 10.4.2. For bead interaction assays and infection assays, an unpaired two-tailed *t*-test was used to assess the significance of results. In addition to this, a Mann–Whitney test was employed to ensure significance as data was not tested for normality and equal variance. All assays were performed in triplicate with statistics based on at least three biological replicates.

## 3. Results

### 3.1. Sequence and Structural Characteristics of IdeC

**Figure 1:**
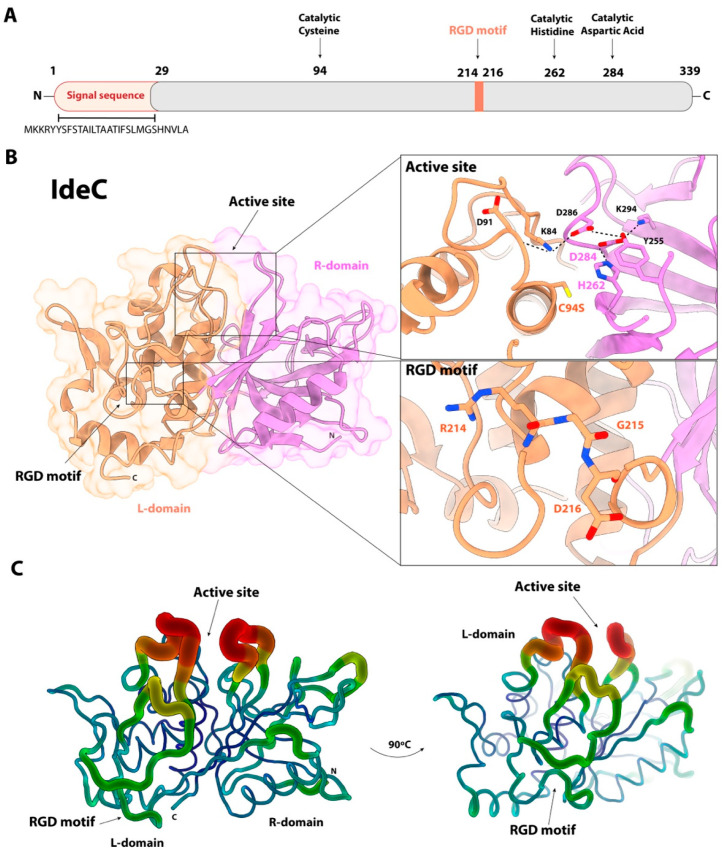
Structure of the IdeC Protein. (**A**) Schematic representation of IdeC; marked are the predicted N-terminal signal sequence (determined using SignalP 6.0 Server), the RGD motif (amino acids 214–216) and the catalytic triad (Cys94, His262 and Asp284). (**B**) The crystal structure of the IdeC protein (PDB code 9HB1) is displayed in both surface and cartoon representations, with the active site and RGD motif highlighted by black arrows. On the right, a detailed view illustrates the IdeC active site and the RGD motif in greater detail. Relevant residues are depicted as capped sticks and labelled. Polar interactions are represented as dotted lines. (**C**) B-factor putty representation of the complete IdeC structure. Regions with lower B-factors are depicted as narrower tubes in shades of blue, indicating highly ordered and tightly packed areas. Wider tubes coloured from orange to red correspond to higher B-factors, highlighting the greater flexibility of the channel that the active site and RGD motif constitute.

**Figure 2:**
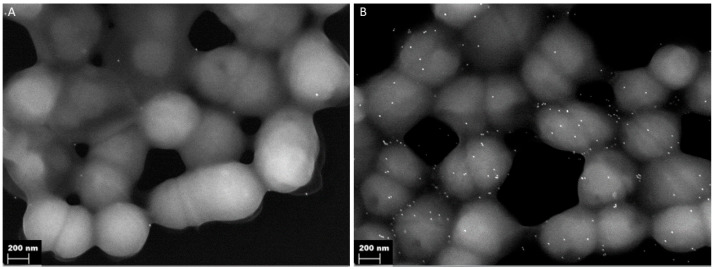
Surface localisation of IdeC. Gold-labelled antibodies detect secreted IdeC on the bacterial surface. (**A**) Binding of gold-labelled antibody without pre-incubation with IdeC-specific antibody served as negative control. (**B**) Binding of gold-labelled antibody after pre-incubation of *S. canis* bacteria with IdeC-specific antibody.

The catalytic properties of IdeC have been investigated in a prior study. We characterised the IgG-cleaving abilities of the protein and determined IdeC to be a species-specific protease cleaving only the IgG of dogs, cats and humans [15]. Here, we investigated the amino acid sequence of IdeC further in order to assess whether there is evidence for a secondary function for the protein. Sequence analysis of the *ideC* gene, using SignalP 6.0, allowed identification of a signal sequence, indicating IdeC is a secreted protein. The sequence MKKRYYSFSTAILTAATIFSLMGSHNVLA was predicted to be the protein’s signal sequence, with the cleavage site located between the Ala29 and Asp30 residues (Figure 1, Supplementary Figure S1). Accordingly, we have classified the amino acids 1–29 as the predicted signal sequence (Figure 1A). Despite being a secreted protein, electron microscopy of *S. canis* using rabbit α-IdeC and α-rabbit-gold-labelled antibodies revealed that IdeC is surface localised (Figure 2).

Based on comparison to the group A streptococcal IdeS protein [13], the amino acids Cys94, His224 and Asp284 were predicted to act as the catalytic triad of the protein. The cys94 residue was then confirmed to be essential for IgG cleavage activity in our previous study [15]. Additionally, an RGD motif was present at position 214–216 (Arg214, Gly215, Asp216) (Figure 1A).

IdeS has been described as resembling the typical 3D structure of the papain superfamily of cysteine proteases, though they do not share significantly similar amino acid sequences [31]. We have previously reported the structural and functional characterisation of IdeC, which also belongs to the Papain-like cysteine peptidase superfamily and is highly structurally, and sequentially, similar to IdeS [15]. The three-dimensional structure of the IdeC cysteine protease presents the active site located at the interface of the left (L-) and right (R-) domains of the protein (Figure 1B), with the catalytic triad (Cys94, His262 and Asp284) involving residues from the two domains (Figure 1A) [15]. Relevant to this study is the position of the RGD motif in the 3D structure of the protein (Figure 1B). The motif is located in the L-domain, positioned away from the catalytic centre and solvent-exposed [15]. It is worth mentioning that the RGD motif is exposed in a region that, together with the active site, presents a high mobility as observed by the distribution of B-factors in the crystal structure (Figure 1C). In this position, the motif could be involved in the recruitment of substrates to the catalytic active site, or interaction with proteins involved in other functions.

The presence of the RGD motif together with the surface localisation of the IdeC protein provides the basis for this study investigating a possible role in adhesion and/or invasion for IdeC.

### 3.2. Replacing the RGD Motif of IdeC with an RGE Motif Does Not Affect Its Enzymatic Activity

**Figure 3:**
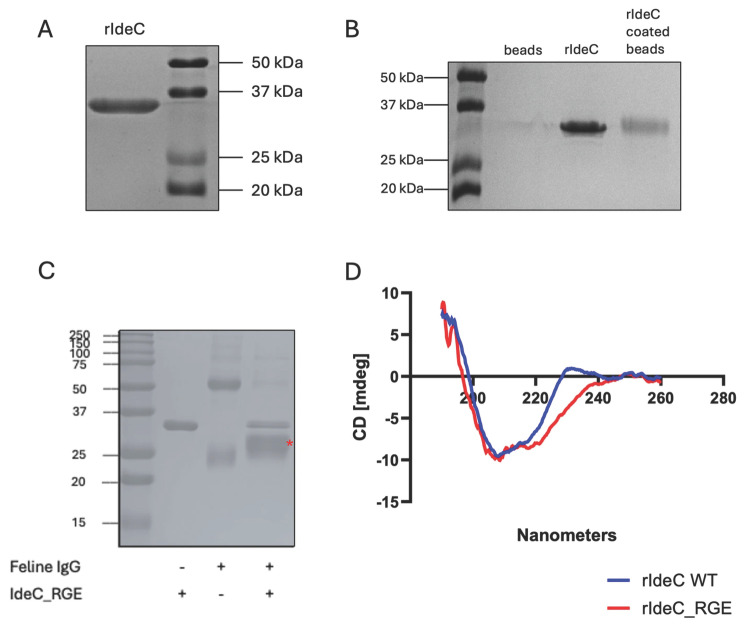
Recombinant expression of IdeC proteins. (**A**) Recombinant IdeC protein visualised as ~35 kDa protein on 15% SDS-PAGE gel. (**B**) SDS-Page detection confirmed that latex beads were successfully coated with rIdeC. (**C**) Feline IgG cleavage assay with rIdeC_RGE_ shows that changing the RGD amino acid motif to a RGE motif still results in IgG cleavage. The cleavage fragment of ~24 kDa is indicated with a red *. (**D**) There is no significant difference in the CD spectra of rIdeC and rIdeC_RGE_.

Two recombinant IdeC proteins were produced for this study: rIdeC wild-type (WT) and rIdeC_RGE_. The recombinant IdeC proteins all consist of residues 30–339 of the IdeC protein, i.e., the protein without the predicted signal sequence, and are ~35 kDa in size (Figure 3A). A single-nucleotide change in the sequence of rIdeC_RGE_ resulted in a glutamic acid replacing the aspartic acid at position 216, resulting in an ‘RGE’ in the place of the RGD motif found at position 214–216. The RGD-to-RGE change has been demonstrated to interfere with integrin-binding without causing structural changes [32]. rIdeC and rIdeC_RGE_ are both capable of IgG cleavage. The cleavage of IgG by IdeC WT has been previously documented [15] and Figure 3C shows that incubation of rIdeC_RGE_ with feline IgG results in an additional band of ~24 kDa which is produced during the breakdown of the heavy chain of the IgG. This indicates that the amino acid change (Asp216Glu) had no effect on the protein’s enzymatic activity. Comparison of the circular dichroism (CD) spectra of both proteins reveals no difference in 3D structure caused by the amino acid change (Figure 3D).

### 3.3. IdeC Interacts with Endothelial and Epithelial Cells

**Figure 4:**
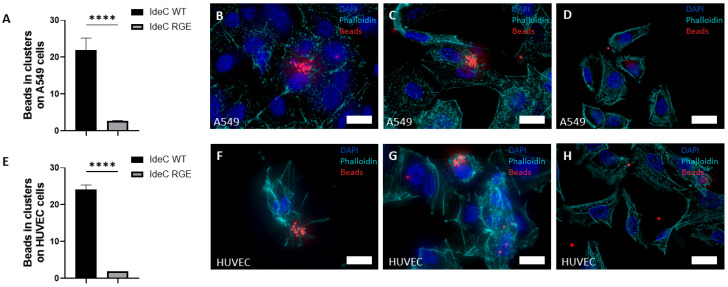
rIdeC-coated beads interact with endothelial and epithelial cells in an RGD-dependent manner. Fluorescent beads were coated with purified recombinant protein and interaction with A549 and HUVECs was microscopically determined after immunofluorescence staining was used; the cells were incubated with 100-fold excess of beads. Graphs demonstrate the average number of beads in clusters associated with A549 (**A**) and HUVECs (**E**). Representative images of bead clusters in A549 (**B**,**C**) and HUVECs (**F**,**G**). Representative images of A549 (**D**) and HUVECs (**H**) with IdeC_RGE_-coated beads. White scale bars indicate 10 µm. Error bars were based on standard deviation from the mean. Statistical significance was assessed using an unpaired two-tailed T test and a Mann–Whitney test. *p*-value of 0.0001 is indicated by ****.

To further define functionalities of IdeC with regard to interaction with eukaryotic cells, we coated fluorescent latex beads with recombinant protein (Figure 3B). Beads were then applied to cell monolayers to investigate the interaction of IdeC with host cells. The fluorescent beads emitted light that was detectable in all fluorescent channels, making differential staining impossible, so internalisation could not be determined by using this assay. However, latex beads coated with WT IdeC showed increased interaction with epithelial monolayers of human adenocarcinomic alveolar basal epithelial cells (A549 cells) (Figure 4A–D) and with human umbilical vein endothelial cells (HUVEC) (Figure 4E–H), compared to BSA-coated beads. rIdeC-coated beads formed clusters in association with both epithelial and endothelial cells; these clusters could be observed 1 h after application of beads but were more common and consisted of more beads at the 2- and 3-h time points post-incubation. The average number of beads per cell in cell-associated clusters was 21 beads in association with the A549 cells and 24 beads with HUVEC (Figure 4A,E).

When these experiments were repeated using rIdeC_RGE_-coated beads, single beads and coupled beads were observed in association with host cells but bead clustering was not observed (Figure 4D,H). Using both the *t*-test and Mann–Whitney test, it was determined that both A549 and HUVECs displayed increased interaction with latex beads coated in rIdeC compared to rIdeC_RGE_ (*p* < 0.0001).

### 3.4. Integrin α_V_β_3_ Mediates IdeC-Uptake

**Figure 5:**
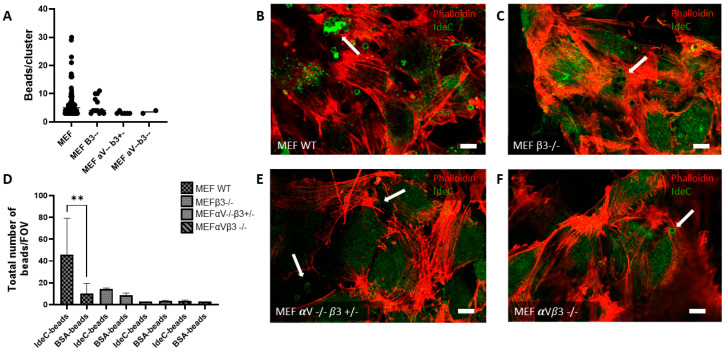
Expression of integrin *α*V*β*3 facilitates increased interaction of rIdeC-coated beads with MEF cells. rIdeC-coated latex beads were applied to MEF monolayers on glass slips. Monolayers were incubated for 3 h before slides were immunostained for imaging. (**A**) Graphical presentation of average number of beads per cluster on MEF cells. (**D**) Graph showing total number of beads interacting with cells, per field of view. The only cell type with a significant difference in interaction between rIdeC-coated beads and BSA-coated beads was the MEF wild-type. (**B**,**C**,**E**,**F**) Representative microscopic image demonstrating MEF cells visualised with phalloidin (red) and rIdeC-coated beads (green); images show interactions with MEF WT cells (**B**), *β*3-integrin-subunit-deleted MEF cells (**C**), *α*V -/- *β*3 +/- integrin-deleted MEF cells (**E**), and on *α*V*β*3-integrin-deleted cells (**F**). White arrows point to beads or clusters of beads; white scale bars indicate 10 µm. Error bars were based on the standard deviation from the mean. Statistical significance was assessed using both an unpaired two-tailed T test and a Mann–Whitney test. A *p*-value of 0.001 is indicated by **.

Results of the cell culture interaction analyses determined that rIdeC-coated beads interact with endothelial and epithelial cells in an RGD-motif-dependent manner. As the RGD motif is notable for being an integrin-binding domain, the function of integrin receptors in the IdeC–cell interaction should be further characterised. For this aim, rIdeC-coated beads were incubated with a series of mouse embryonic fibroblast (MEF) cell lines, which were used for these experiments, with either single- or double-subunit deletions for the integrin α_V_β_3_. Cell lines used were the WT MEF cells along with MEF β_3_ -/-, MEF α_V_ -/-β_3_ +/- and MEF α_V_β_3_ -/-. In the WT MEF cells, we observed the same clustering of rIdeC-coated beads (Figure 5A) as was demonstrated in A549 and HUVECs (Figure 4).

The size and frequency of these clusters were reduced in the β3 subunit deletion (Figure 5C), and further reduced in the MEF α_V_ -/-β_3_ +/- and MEF α_V_ -/-β_3_ -/- double-subunit deletion (Figure 5E,F). Figure 5A shows the range in size of bead clusters observed in each of the cell lines; the WT MEF cells are the only cells where clusters with more than 10 beads were observed. Figure 5D shows a side-by-side comparison of rIdeC-coated beads and BSA-coated beads for each cell line. The only cell line where there is a significant difference in the interaction of rIdeC-coated beads compared to BSA-coated beads is the MEF with WT integrin expression.

### 3.5. IdeC Plays a Role During S. canis Infection

**Figure 6:**
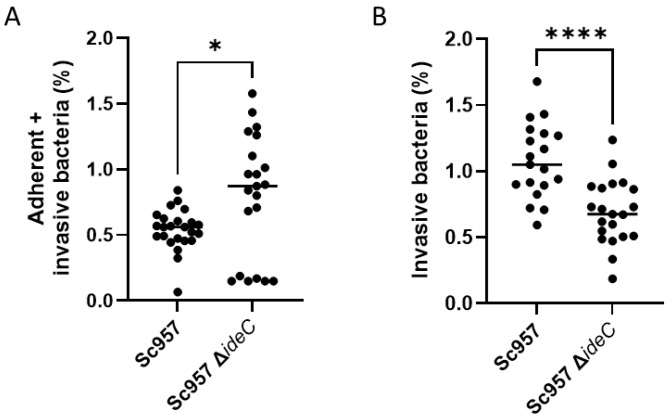
Function of IdeC in bacterial adhesion and invasion of A549 cells. A549 cells were infected with wild-type Sc957 and Sc957ΔideC at an MOI of 10. (**A**) Adhesion samples: After 2 h of bacterial infection, cells were lysed, and the CFU/mL as a percentage of the CFU/mL of the inoculum was calculated. (**B**) Invasion samples: After 2 h of bacterial infection, cells were incubated for a further 45 min in medium supplemented with erythromycin before being treated as described previously. Graphs show data from three biological replicates. Statistical significance was assessed using an unpaired two-tailed *t*-test and a Mann–Whitney test; a *p*-value of 0.05 was considered significant and indicated by *, and a *p*-value of 0.0001 is indicated by ****.

The preceding results demonstrate that IdeC can facilitate adhesion and uptake of latex beads into HUVECs and A549 and MEF cells. However, to state concretely whether IdeC plays an active role in cell culture infection, an *ideC* gene-deleted *S. canis* mutant named Sc957Δ*ideC* was generated. There was no significant difference in growth rate between the WT and the deletion mutant (Supplemental Figure S2), although in liquid culture, the Sc957Δ*ideC* strain appeared to aggregate more than the WT. In cell culture infection analyses with human epithelial A549 cells, a significant reduction in the percentage of internalised Sc957Δ*ideC* bacteria was observed when compared to infection with the WT strain (Figure 6B).

**Figure 7:**
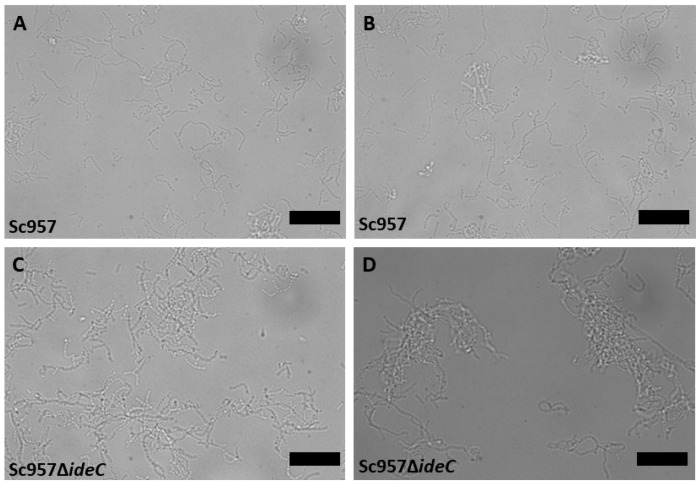
Representative light microscopy images displaying differences in bacterial aggregation of Sc957 and the Sc957Δ*ideC*. (**A**,**B**) Images of Sc957 wild-type. (**C**,**D**) Images of Sc957Δ*ideC*. Black scale bar signifies 10 µm.

However, significantly more Δ*ideC* streptococci interacted with A549 than WT *S. canis* overall (Figure 6A). There was also a much broader range of results when considering the mutant, compared to the WT. It is possible that this broader range of results is due to the increased aggregation displayed by the Δ*ideC* mutant, so microscopy was employed to visualise any differences in the interactions/appearance of the bacteria (Figure 7). This confirmed that Sc957Δ*ideC* displays increased self-interaction among bacteria (Figure 7C,D), compared to the WT (Figure 7A,B). This increased bacterial interaction observed in the mutant may be responsible for the formation of bacterial aggregates.

## 4. Discussion

Characterisation of virulence determinants in *S. canis* has thus far been limited, although there have been studies that have identified genes in *S. canis* that are homologues of virulence genes reported in other bacteria [5,6]. In our previous study, we have identified IdeC as a cysteine protease that cleaves feline, canine and human IgG [15]. The protein sequence of IdeC is highly similar to that of IdeS of *S. pyogenes* and the protein is predicted to function in accordance with the mechanism described by Von Pawel-Rammingen and colleagues—in short, as a secreted protein that cleaves IgG that is bound to the bacterial surface via M-like proteins or other surface antigens [13]. Among the similarities shared by IdeS and IdeC are the putative catalytic triad and the presence of an RGD motif (Figure 1). The presence of this motif is a feature of proteins that interact with integrins, including a variant of streptococcal pyrogenic exotoxin B (SpeB) and also *S. pyogenes*, a cysteine protease that has been shown to directly interact with cell surface integrins as well as to degrade ECM proteins [33,34].

‘Moonlighting proteins’ are proteins that exhibit more than one biological function, often involved in one function as a secreted protein, and another when attached to the cell wall [35]. The first ‘moonlighting’ protein identified was a *S. pyogenes* glyceraldehyde 3-phosphate dehydrogenase (GAPDH) with multiple-bingeing activity, which is essential for virulence [36,37]. Another important streptococcal ‘moonlighting’ protein is enolase, with roles in glycolysis and also in plasminogen binding [38]. We have described IdeC here as a secreted protein that can bind to the bacterial surface after secretion (Figure 1 and Figure 2). Since its function as a secreted IgG protease has been described previously, here we focus on a possible second/’moonlighting’ function it may perform as a surface-bound protein. For this purpose, the previously reported protein structure was re-examined. The crystal structure revealed the location of the RGD motif of the protein, positioned on an accessible, outward-facing loop (Figure 1B). Other streptococcal proteins have been described with RGD motifs that have been proven to interact with integrins via the RGD residues; for example, the mSpeB2 variant of SpeB interacts with the integrin α_V_β_3_ and the IdeS/Mac-1 variant Mac5005 binds both α_V_β_3_ and α_IIb_β_3_ integrins [26,39]. In both proteins, the RGD is located on a loop, in SpeB on a surface loop near the C-terminal protease domain [40] and in IdeS on a loop in the N-terminal domain [41]. The positioning of the RGD on a loop removed from the catalytic centre of the protein suggests it is possible for the protein to interact with cell surface proteins while maintaining its IgG-cleaving action.

A surface localised protein with an integrin-binding site is an interesting candidate to investigate for a role in adhesion to or invasion of eukaryotic cells, which can serve as a prerequisite for colonisation and tissue distribution. Streptococcal infections usually begin with the interaction of bacteria with host cells via adhesins [42]. Stockbauer et al. reported that though the SpeB variant containing the RGD motif was not exclusively capable of causing invasive disease, M-types with the binding site were frequent originators of severe disease. Notably, the M1 *S. pyogenes* serotype expressed the RGD-containing variant of SpeB [39]. The M1 serotype is strongly associated with invasive disease [43]. We specifically show here that the integrin α_V_β_3_ interacts with IdeC, and can facilitate the RGD-dependent interaction of IdeC with host cells (Figure 4 and Figure 5). The integrin α_V_β_3_ was chosen here as it had previously been shown to interact with streptococcal proteins containing the RGD motif [26,39], and is present in both A549 and HUVECs; however, this does not mean that α_V_β_3_ is the only integrin capable of interaction with IdeC. The α_V_ subunit has specifically been shown to promote *Streptococcus agalactiae* invasion of epithelial cells [44]. Indeed, Figure 5C shows that deletion of only the β_3_ subunit is not sufficient to interfere with IdeC binding to MEF cells; the binding of IdeC with other α_V_ subunit-containing integrins could therefore be an interesting avenue to pursue in clarifying the mechanism of IdeC.

Though we show here that IdeC is capable of interacting with host cells, it is not expected to be the only factor mediating adhesion and invasion of *S. canis* to host cells. We see in Figure 6 that there is still adherence to and invasion into A549 cells by bacteria when *ideC* is deleted. Indeed, deletion of *ideC* has different implications for adherence and invasion. Figure 6A shows almost a doubling in the combined adhesive and invasive fractions in the Δ*ideC* mutant, while the invasion alone (Figure 6B) is reduced significantly. We can infer from this that the increase we see in the adherent and invasive fractions combined is mainly due to an increase in external adherence only.

From this it seems that the deletion of *ideC* promotes adhesion. However, when we consider the increased aggregation exhibited by the *ideC*-negative strain (Figure 7), it is possible that much of the observed increase in ‘adherence’ is actually due to increased aggregation exhibited by this strain; i.e., the deletion of *ideC* results in increased interaction with other bacteria and not increased adhesion to the host cell. It is possible that the decrease in invasion observed in the Δ*ideC* strain results from a decrease in bacteria actually interacting with the A549 cells or an inability of the cells to take up large aggregates of bacteria. It has been demonstrated in *S. pyogenes* that bacterial aggregation inhibits uptake by phagocytic cells [45]. When we consider that the RGD–integrin interaction has been demonstrated to be crucial for the integrin clustering that leads to signal activation, allowing invasion of pathogens into host cells [19,20], it is also possible that the absence of the RGD motif of IdeC limits the invasive ability of the bacteria despite their ability to adhere to host cells via an IdeC-independent mechanism.

The aggregation phenotype demonstrated in Figure 7 cannot be conclusively explained based on this study, though possible mechanisms can be suggested. It is possible that the absence of IdeC results in the increased availability of another surface protein of *S. canis*, which is responsible for the binding of IdeC to the bacterial surface after secretion. Such a protein could theoretically form bonds with other surface-associated proteins when not bound to IdeC, resulting in aggregation. Recent work in *S. pyogenes* has demonstrated that *S. pyogenes* nuclease A (SpnA) is involved in IdeS binding to the bacterial surface, although it was determined that it was not the sole binding partner of IdeS on the bacterial surface [46]. This may indicate that IdeC also has multiple binding partners responsible for its surface association, and these may be capable of interaction. The M-like protein of *S. canis* is a plasminogen-binding protein that is covalently bound to the cell surface [8]. M proteins are antiphagocytic proteins that bind to a number of different host proteins, e.g., fibrinogen [47]. SCM has been shown to form homophilic interactions with itself that can cause bacterial aggregates [4]. It is possible that IdeC regulates the amount of surface-exposed SCM. IdeC-specific gene deletion may remove regulation of SCM, leading to increased levels of SCM on the bacterial surface. A deeper investigation into the interactions of IdeC with the bacterial surface should be carried out to explain the aggregation phenotype, as well as the ability of IdeC to bind back to the bacterial surface after secretion (Figure 2); these interactions could be reliant on one surface protein like ScM or involve several.

Moreover, results depicted in Figure 6 demonstrate that a greater number of WT *S. canis* bacteria are in the invasive-only fraction compared to the combined fraction of adherent and invasive bacteria. As these fractions are measured 40 min apart, this may indicate that *S. canis* is replicating within the host cell after invasion. When bacteria invade host cells they are initially taken up into membrane-bound phagosomes. Some bacteria have evolved strategies to escape these phagosomes and survive intracellularly. As a consequence, they can multiply and potentially spread to adjacent cells [48]. In this respect, other streptococci have been demonstrated to propagate successfully intracellularly and this has been linked to immune evasion and persistent infection. For example, *S. pyogenes* is able to modulate the human macrophage via the M1 protein to promote persistence within vacuoles [49]. In line with this, another study reported that *S. pyogenes* was shown to evade the host immune response by surviving within phagocytic cells [50].

## 5. Conclusions

In conclusion, in addition to the formerly described role of IdeC as an IgG-cleaving protease [15], the internalisation into epithelial and endothelial cells might represent a second important immune evasion mechanism. The presented data provides strong evidence that IdeC does indeed play a role in internalisation of *S. canis* into host cells, thereby representing a ‘moonlighting’ function important for the pathophysiology of *S. canis*. Furthermore, the cellular uptake into host cells depends on the interaction of the RGD motif with integrins on the host cell surface, representing a pivotal molecular pathomechanism mediated by the cysteine protease IdeC of *S. canis*. This discovery is especially significant given how few virulence determinants have been described in *S. canis*, and how little we understand about the dissemination of *S. canis* throughout the host that can lead to invasive disease.

This study provides important evidence for a function of IdeC outside of IgG cleavage; however, it also highlights several avenues where further research is required to fully understand the role this protein plays during infection. Notably, the interaction of IdeC with other proteins, including, as discussed above, other integrins and proteins present on the bacterial cell surface, should be explored in further work.

## Data Availability

The raw data (including bead and bacterial count numbers associated with bead association and S. canis infection assays) presented in the study are openly available in FigShare at DOI: https://doi.org/10.6084/m9.figshare.30305872.

## Supplementary Materials

Supplementary figures and raw data are openly available at figshare. Supplementary figures are available under DOI: 10.6084/m9.figshare.30306004 (https://figshare.com/articles/figure/Supplementary_Figures/30306004, accessed on 12 April 2026) [51]. Raw data, including bead and bacterial count numbers associated with bead association and *S. canis* infection assays, are available under DOI: 10.6084/m9.figshare.30305872 (https://figshare.com/articles/dataset/Supplementary_raw_data/30305872, accessed on 12 April 2026) [52].
